# Healthcare professionals’ acceptance of BelRAI, a web-based system enabling person-centred recording and data sharing across care settings with interRAI instruments: a UTAUT analysis

**DOI:** 10.1186/1472-6947-13-129

**Published:** 2013-11-27

**Authors:** Dirk Vanneste, Bram Vermeulen, Anja Declercq

**Affiliations:** 1 Elderly Care Research Unit at LUCAS, KU Leuven, Kapucijnenvoer 39, 3000 Leuven, Belgium; 2 Elderly Care Research Unit at LUCAS and Center for Sociological Research, KU Leuven, Kapucijnenvoer 39, 3000 Leuven, Belgium

**Keywords:** BelRAI, InterRAI, Older people, Person-centred, Computerisation, Data sharing, Integrated care, UTAUT, Technology acceptance, SEM, PLS

## Abstract

**Background:**

Healthcare and social care environments are increasingly confronted with older persons with long-term care needs. Consequently, the need for integrated and coordinated assessment systems increases. In Belgium, feasibility studies have been conducted on the implementation and use of interRAI instruments offering opportunities to improve continuity and quality of care. However, the development and implementation of information technology to support a shared dataset is a difficult and gradual process. We explore the applicability of the UTAUT theoretical model in the BelRAI healthcare project to analyse the acceptance of the BelRAI web application by healthcare professionals in home care, nursing home care and acute hospital care for older people with disabilities.

**Methods:**

A structured questionnaire containing items based on constructs validated in the original UTAUT study was distributed to 661 Flemish caregivers. We performed a complete case analysis using data from 282 questionnaires to obtain information regarding the effects of performance expectancy (PE), effort expectancy (EE), social influence (SI), facilitating conditions (FC), anxiety (ANX), self-efficacy (SE) and attitude towards using technology (ATUT) on behavioural intention (BI) to use the BelRAI web application.

**Results:**

The values of the internal consistency evaluation of each construct demonstrated adequate reliability of the survey instrument. Convergent and discriminant validity were established. However, the items of the ATUT construct cross-loaded on PE. FC proved to have the most significant influence on BI to use BelRAI, followed by SE. Other constructs (PE, EE, SI, ANX, ATUT) had no significant influence on BI. The ‘direct effects only’ model explained 30.8% of the variance in BI to use BelRAI.

**Conclusions:**

Critical factors in stimulating the behavioural intention to use new technology are good-quality software, interoperability and compatibility with other information systems, easy access to computers, training facilities, built-in and online help and ongoing IT support. These findings can be used by policy makers to maximise the acceptance and the success of new technology. For researchers, the conclusions of the original UTAUT study with regards to the item and scale construction should not be copied blindly across different information systems. A bottom-up approach is preferred when building upon the UTAUT model.

## Background

The 21st century is the century of the ‘ageing population’. In Belgium (population 2011: 11 million), there are now 1.88 million people aged 65 and older which equals 17% of the population [1]. In 2020, they will be 2.22 million, which is plus 20%. Over the next 10 years the number of people over 80 will grow to nearly 578,500, compared with 479,000 in 2011 [2].

As people live longer, healthcare environments are increasingly confronted with older persons, characterised by chronic conditions and/or comorbidities necessitating long-term care [3-6] and rising costs. There is also a substantial shift away from institutional caregiving. This evolution increases the complexity of caregiving since multiple service providers are delivering care to individuals [3,7,8]. The need to receive support from multiple service providers has important implications for persons with complex care needs [9].

As these people transfer between settings, accurate communication and sharing of crucial information is fundamental in providing high quality care [10]. A lack of information (transfer) can result in errors [11], increased assessment burden, and frustration among care recipients and their supporting network [12]. Deficient information transfer or communication can result in uncoordinated care, adverse events and iatrogenic complications influencing morbidity, mortality and hospital outcomes [10,11]. It appears that clinical information systems that typically have been designed to support single service providers in one setting no longer meet the necessary requirements [8].

As people migrate through this maze of healthcare providers, the need for integrated and coordinated assessment systems providing comparable information increases [13], as does the need for service provisions that overcome barriers [3,8,14-16]. The importance of good communication in healthcare environments [17] to enable person-centred care and increase safety cannot be emphasised enough. Collected data must be available and useful to those who have to decide at the personal, clinical, managerial, and public policy levels [3,18]. In complex healthcare and social care systems in which people with the same needs might receive different services in different sectors and in which changes in one subsystem might affect other subsystems, thinking at the system level is crucial. To this end, it is necessary to have an integrated, standardised and computerised collection of information in a language that is clear and understandable to all users [3,8,19,20].

The interRAI suite of instruments [21] addresses the changing strengths, preferences and needs of vulnerable people rather than the organisation which provides services at a single point in time [6,8]. This ‘third generation’ multi-domain suite of compatible instruments has the potential to transfer and share high quality person-centred information and to compare people, services and outcomes across settings [12,22-26].

Although these instruments provide opportunities to improve continuity of care, as well as efficiency and quality of care [27-37], they cannot reach their full potential with paper-based recording only. Paper-based recording has severe limitations: it is applicable only to slow, manual processing, it is difficult to share, it may lack information concerning the history of treatment, it is centred upon institutional boundaries, it is vulnerable to errors, it lacks interoperability with other information systems and its information may not be presented in ways relevant to specific care contexts [38]. Moreover, the complex algorithms of the interRAI suite of instruments that generate decision support outcomes and guidelines are highly dependent on automation.

These problems can only be addressed by the use of computer-based information technologies [8,19]. Information technology (IT) is already being used to prevent human error in healthcare and to improve patient care outcomes worldwide. In 2000, the Institute of Medicine in Washington DC recommended increasing efforts to incorporate information technology into the delivery of patient care [39].

In Belgium, web-based software has been developed to support the use of the interRAI instruments in different settings and in different languages^a^. This software has been tested and evaluated. As, to our knowledge, there is no evidence of a fully computerised set of interRAI suite instruments throughout the whole world, this research can be considered innovative.

However, developing and implementing information technology to support a shared dataset is a difficult, slow and gradual process [40]. There is also a gap between the two different professional approaches. On the one hand, information and communication technologies (ICT) scientists may have the technical skills to develop an application, but they are unlikely to fully understand how the application must operate in order to support the organisation. On the other hand, healthcare organisations may be able to identify and express the ‘what’, ‘when’, ‘for whom’ and ‘where’ issues concerning an application to support their goals, but they may not have the technical skills to make it happen [41]. Consequently, in order to achieve a successful implementation, substantial challenges have to be tackled [42]. There is a need for good-quality software, ongoing IT support, access to computers, and a degree of computer literacy in care organisations [8,19,43]. Moreover, there are numerous possible reasons for why change may be opposed: misunderstandings about the need for change, fear of failure, fear of loss of professional autonomy, fear of additional workload, fear of transparency, reluctance to share instrumentation [8], concerns about confidentiality, concerns about the impact on human resources and the professional demands that come with such a change. In addition, the ICT professionals need to have a grasp of what is actually happening and needed in care practice.

With all this in mind, caregivers will not necessarily accept a (new) health information system and use it in daily practice [16,44]. Information technology that is not used, or is only partly used, cannot reasonably be expected to contribute to improving the quality of care. On the contrary, it can be an extra burden or cause other problems [45]. It is clear that the impact of such a change could be a contributing factor leading to an organisational crisis, causing conflict among professional groups. It is therefore crucial to understand how aspects of real-world activities relate to one another [41] and to clarify what the influencing factors are of the acceptance of and the use or failure of the information system.

In this paper we explore the applicability of the unified theory of acceptance and use of technology (UTAUT) theoretical model in the BelRAI healthcare project in analysing the acceptance of the BelRAI web application by healthcare professionals in three Belgian care settings (home care, nursing home care and acute hospital care for older people with disabilities). This will help to broaden our understanding of the development and implementation of healthcare information systems.

### The unified theory of acceptance and use of technology

Information technologies permeate all aspects of human life and today’s organisations, having an increasing importance [46]. With respect to understanding individual acceptance of new information technologies or systems, it is not surprising that prior research was mainly focused on sociological and psychological factors [45-48]. Understanding and clarifying the drivers influencing the acceptance and the use or failure of information systems (IS) has always been a long-term issue in technology and IS research [49]. To promote a unified view of user acceptance and in an effort to identify the most significant influences, Venkatesh et al. [46] reviewed and compared the following behavioural models (evolved models not included): social cognitive theory (SCT) [50], theory of reasoned action (TRA) [51], theory of planned behaviour (TPB) [52,53], model of PC utilization (MPCU) [54], technology acceptance model (TAM) [49], combined TAM and TPB (C-TAM-TPB) [55], innovation diffusion theory (IDT) [56] and motivational model (MM) [57]. Each of these models has the intention to use technology or the actual use of technology as the dependent variable whereby the variance in user intentions is explained between 17% and 53% [46].

By integrating the conceptual and empirical similarities of these eight models, UTAUT explains up to 70% of the variance in intention to use technology and about 50% of the variance in technology use [46]. This makes UTAUT an interesting starting point from which to understand technology or system acceptance.

**Figure 1:**
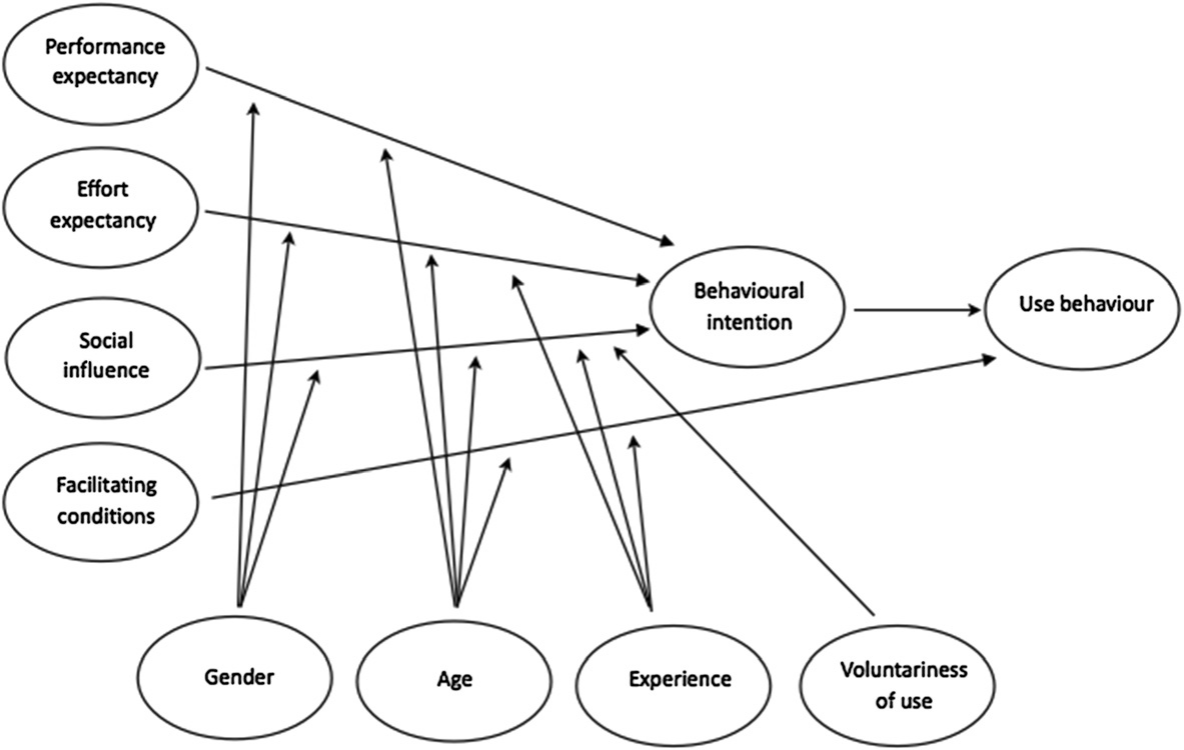
**Original UTAUT model **[46]**.**

According to Venkatesh et al. [46], UTAUT (Figure 1) includes three direct determinants of behavioural intention to use a system:

•performance expectancy (PE) defined as the degree to which an individual believes that using the system will help him or her to achieve increases in job performance. Performance expectancy proves to be a significant determinant of behavioural intention, moderated by gender and age such that the effect is strongest for younger men;

•effort expectancy (EE) defined as the degree of ease associated with the use of the system. It was found that the effect of effort expectancy on behavioural intention is moderated by gender and age such that the effect is strongest for older women in the early stages of experience with the system;

•social influence (SI) defined as the degree to which an individual perceives that important others believe he or she should use the new system. It was found that the effect of social influence on behavioural intention is moderated by gender, age, experience and voluntariness such that the effect is strongest for older women in early stages of experience in mandatory contexts.

UTAUT contains also two direct determinants of use behaviour:

•facilitating conditions (FC) defined as the degree to which an individual believes that an organisational and a technical infrastructure exists to support use of the system. It was found that the effect of facilitating conditions on use behaviour or system use is moderated by age and experience such that the effect is strongest for older workers in later stages of experience;

•behavioural intention (BI) is not defined explicitly but consistent with the underlying theory for all of the intention models, Venkatesh et al. [46] have found that behavioural intention has a significant positive influence on use behaviour or system use. They state: ‘The role of intention as a predictor of behaviour (e.g., usage) is critical and has been well-established in IS and the reference disciplines.’

Finally, Venkatesh et al. [46] describe four moderators that would influence the effect of the determinants on behavioural intention and/or use behaviour: gender, age, experience with the system and voluntariness of use.

## Methods

### Research model and hypotheses

UTAUT was originally developed outside of the healthcare context. We built upon this framework to complement prior research on technology acceptance and use [45,48,58,59] by exploring the applicability of the UTAUT model in the context of the BelRAI healthcare project. The study has the following characteristics.

Firstly, as there are no reliable indicators available on actual use of the BelRAI web application (e.g., archival system logs providing information on duration of use, number of features used, frequency of use of each feature, …), the emphasis of the study is on understanding which determinants influence behavioural intention to use the system. Consequently, we could not test the influence of facilitating conditions on actual usage.

Secondly, organisational contexts can increase or decrease the variation in phenomena and result in relationships fluctuating from stronger to weaker, positive to negative, significant to non-significant [60,61]. Consistent with this, healthcare professionals might respond differently than people in other types of organisations.

Previous research in different healthcare settings [45,62-64] mentioned that performance expectancy or related constructs strongly influence behavioural intention. The uptake of technologies that was perceived as advantageous (financially, or with respect to time-saving and greater job satisfaction) was more straightforward for clinicians, nurses or other healthcare professionals. Following this logic, we believe that performance expectancy will have an influence on behavioural intention in this study context (H1, Table 1).

**Table 1:** Hypotheses

	
**H1**	Performance expectancy will influence behavioural intention to use BelRAI.
**H2**	Effort expectancy will influence behavioural intention to use BelRAI.
**H3**	Social influence will influence behavioural intention to use BelRAI.
**H4**	Facilitating conditions will influence behavioural intention to use BelRAI.
**H5**	Anxiety will influence behavioural intention to use BelRAI.
**H6**	Self-efficacy will influence behavioural intention to use BelRAI.
**H7**	Attitude towards using technology will influence behavioural intention to use BelRAI.

Wills et al. [45] and de Veer et al. [63] found that an easy-to-use technology enhanced the uptake of a new system among caregivers. Hence, we expect that effort expectancy will have an influence on behavioural intention (H2).

Consistent with Venkatesh et al. [46], Wills et al. [45] found that social influence constructs also became significant in mandatory healthcare settings particularly among women in early stages of experience. This research is conducted in a mandatory setting with mostly female caregivers. We suggest that social influence will have an impact on behavioural intention (H3).

De Veer et al. [63] suggested that the acceptance of a new technology by nurses was influenced by facilitating characteristics. Chau et al. [64] found that constructs related to facilitating conditions influenced behavioural intention in healthcare settings. Therefore, we have reason to suspect that facilitating conditions will have an influence on behavioural intention to use the BelRAI web application (H4).

Anxiety (ANX), self-efficacy (SE) and attitude towards using technology (ATUT) are determinants in their original models. Based on SCT, anxiety is defined as evoking anxious or emotional reactions when it comes to using a system, and self-efficacy is defined as judgement of one’s own ability to use a system. Based on TRA, ATUT is defined as an individual’s overall affective reaction to using a system. Having no direct effect on behavioural intention, ANX, SE and ATUT were dropped in Venkatesh’s study [46]. However, we thought it would be valuable to re-examine the influence of these constructs on behavioural intention in the BelRAI healthcare setting (H5, H6, H7).

Thirdly, as we have not examined the potential moderating roles of age, gender, experience with the system and voluntariness, we have restricted our study to direct effects only. Moreover, Venkatesh et al. [58] found that a more parsimonious version of UTAUT, with only age as a moderator, performs better in explaining behavioural intention among doctors in a hospital setting.

### Settings and context

The study focuses on data derived from the BelRAI feasibility project integrating home care (interRAI home care assessment instrument [65]), residential or nursing home care (interRAI long-term care facility assessment instrument [66]) and acute hospital care (interRAI acute care assessment instrument [67]) for (frail) older people [68]. We have also included data of a study about the effects of innovative home care projects (alternatives or add-ons to the standard home care services) in Flanders (Belgium) using the interRAI home care assessment instrument [69].

### Participants

The participants are a heterogeneous group of Flemish licensed or qualified caregivers (nurses, physical therapists, occupational therapists, speech-language therapists, dieticians, podiatrists, social workers, physicians, psychologists, dentists, pharmacists). They were all using the BelRAI web application for interRAI assessments for the duration of the project in their own (home, residential or acute hospital) care setting on a mandatory basis. All the participants attended training sessions customised to their specific needs and all persons who followed a training session were asked to participate in this study.

### Questionnaire

A Flemish-Dutch translation of a structured questionnaire for data collection was used, containing items based on constructs validated in the original UTAUT study [46]. Results from an earlier study have shown that the UTAUT tool is ‘robust enough to withstand translation and to be used cross-culturally, outside its original country and language of origin’ [70]. The original English version of the UTAUT tool was translated independently into a Flemish-Dutch (official language of the Northern region of Belgium) version by a qualified (licensed) translator on an item-by-item basis using the back-translation process. Thereafter, two bilingual speakers carefully reviewed the translation ensuring that meaning and nuance were not lost and remained as true to the original as possible [71]. Changes only relate to the name of the system used.

The questionnaire items related to the UTAUT constructs^b^ were measured by means of a 1 to 7-point Likert agreement scale ranging from “Strongly disagree” to “Strongly agree”. Effort expectancy is reverse coded so that high effort expectancy suggests high ease of use [46].

In the questionnaire additional information such as age and gender was also collected.

### Data collection

The questionnaire and a cover letter were designed using QuestionPro [72] and made available to the participants on the web at one point in time. The participants were invited by email. To maximise the response rate and reduce non-response, non-responders were sent a reminder requesting participation one week after the initial-mail-out and again after three weeks [73].

### Ethical considerations

As this study occurred without patient involvement, no approval by an ethics committee was required in Belgium. Participants were assured response anonymity.

### Data analysis

Theoretical concepts such as attitudes, intentions and preferences, also known as latent variables, factors or constructs, cannot be observed or measured directly [74,75]. Constructs are measured indirectly through indicators (observable, measurable or manifest variables such as an item on a questionnaire) designed to elicit responses related to a construct [76]. Scaling techniques have been developed to study the complexity inside a system and to derive information on constructs from indicators. However, to overcome limitations of the first generation regression-based approaches, more and more researchers started using structural equation modelling (SEM) as an alternative [77].

In this study, data analysis was done using SEM. SEM is a multivariate technique combining aspects of multiple regression and factor analysis and thus testing underlying factors and hypotheses simultaneously in the same analysis [76,78]. In an SEM analysis researchers estimate a causality network according to a theoretical model, linking multiple dependent or independent constructs, each measured through a number of indicators, testing theory with empirical data [79]. More specifically, there are two approaches, the covariance-based (e.g. linear structural relations a.k.a. LISREL being the most popular one among social science researchers) and the variance-based or components-based approach (e.g. partial least squares a.k.a. PLS being widely used in performing information systems research) [45,46,48,58,59,76,77,80]. Covariance-based SEM examines all possible specified and unspecified covariances in the model. PLS only examines the explicitly stated covariances in the model [76] and focuses on maximising the variance of the dependent variables which are explained by the independent ones [77].

The PLS path modelling approach was reported in the original UTAUT study [46] as having minimal demands on sample size, measurement scales and residual or underlying data distributions [81,82].

Like any SEM, PLS refers to two inter-related models [76,78]: a measurement or outer model describing the constructs (latent variables) that make up the model and the relationships between the constructs and their indicators (manifest variables) and a structural or inner model describing the causal relationships among these constructs [74,79].

Specifically, for PLS, the generated weights and loadings are outer model parameter estimates, while the path coefficients are inner model parameter estimates.

To evaluate the measurement model, PLS performs a confirmatory factor analysis (CFA) by testing the mandatory [75] reliability and construct validity (factorial validity of the constructs). Reliability relates to the measurement within constructs and is thus independent of the status within other constructs; construct validity relates to the measurement between constructs [75,80].

The evaluation of the internal consistency of constructs (ICR) (e.g. Cronbach’s alpha (CA) to test the unidimensionality of a set of questionnaire items measuring the extent to which all the variables are related to each other) is one technique to assess reliability. Moreover, PLS analysis provides other coefficients attesting to the reliability of the survey instrument such as composite reliability (CR) and average variance extracted (AVE) for each construct. As a rule of thumb the cut-off for (CA) is 0.70 [83,84]. The recommended respective thresholds for CR and AVE are 0.70 and 0.50 [85].

To establish construct validity [75], PLS examines convergent and discriminant validity of the scale estimating how well a variable measures what it is intended to measure [45] or how well the measurement items relate to the constructs [80].

Convergent validity is established when each measurement item of the model loads with a t-value above 1.96 (rejecting the null hypothesis of no effect) on its latent construct meaning that each measurement item correlates strongly with its theoretical pre-specified construct. The p-value of this t-value should be significant at least at the 0.05 alpha protection level [80].

Discriminant validity is established under two conditions: 1) the correlation of the latent variable scores with the measurement items needs to show an appropriate pattern of loadings, one in which the measurement items load highly (> 0.70) on their theoretically assigned construct and weakly (< 0.30) on other constructs (cross loadings); 2) establishing discriminant validity in PLS also requires an appropriate AVE analysis [75,80]. With the latest version of PLS-Graph 03.00 build 1126 of 2003, AVEs are generated automatically using bootstrapping as a resampling procedure. The AVE measures the variance captured by a latent construct being the shared (or explained) variance. The square root of AVE of each construct should be much larger than its variance shared with any of the other constructs in the model and should be at least 0.50. Conceptually, it is equivalent to saying that the correlation between the construct and its measurement items should be larger than its correlation with the other constructs [45,76,80].

Having established reliability, convergent and discriminant validity of the constructs, the next step is to test the structural model for the hypothesised paths. To evaluate the structural model, PLS estimates path coefficients for each hypothesised path using bootstrapping, a non-parametric technique for assessing the precision of the PLS estimates [81,86]. The corresponding t-values suggest significance of the coefficients (t-values > 1.96, significance level p < 0.05) [80]. To ascertain how well the model fits the hypothesised relationship, PLS generates the square of the correlation coefficient (R^2^) for each dependent construct in the model. Similar to regression analysis, R^2^ is interpreted as the percentage of shared (or explained) variance and thus represents the proportion of variance in the dependent constructs which can be explained by the independent ones [45,77].

We ran PLS-Graph ©, a software package which applies PLS [74,81,86] for SEM. Statistical analysis system (SAS) 9.3 © was used for descriptive analysis and creation of PLS-Graph data files.

## Results

### Sample characteristics

Of the 661 invitations to take part in the study, 601 questionnaires were viewed by the persons invited and 421 were started. 282 respondents fully completed the questionnaire. 216 of them (76.60%) were female. 69 (24.47%) were between the ages of 20–29, 85 (30.14%) between 30–39, 68 (24.11%) between 40–49 and 60 (21.28%) were older than 50. 80.14% of the participants had been using the new BelRAI application for more than 4 months; 70.21% had been using the technology for more than 7 months.

With PLS we performed a complete case analysis using data of 282 questionnaires.

### The measurement model

To evaluate the measurement model, PLS tests the reliability and the construct validity. Reliability relates to the measurement within constructs. Construct validity relates to the measurement between constructs [75,80].

As in the original study of Venkatesh et al. [46], for practical analytical reasons the constructs were operationalised by using the highest-loading items^c^ from each of the respective scales (Table 2). Given this specific healthcare situation, these items did not always accord with the highest-loading items used to measure the core constructs in the original UTAUT: only the items with an asterisk were selected for inclusion in the final UTAUT model in the study of Venkatesh et al. [46].

**Table 2:** The measurement model: individual loadings of the highest-loading items in this study

**Construct**	**Individual item**	**Measure**	**PLS item loading (N = 282)**
**PE**	U5	Using BelRAI makes it easier to do my job.	0.9384
	RA2	Using BelRAI improves the quality of the work I do.	0.9604
	RA3	Using BelRAI makes it easier to do my job.	0.9618
	RA4	Using BelRAI enhances my effectiveness on the job.	0.9700
	RA5*	Using BelRAI increases my productivity.	0.9506
**EE**	EOU1	Learning to operate BelRAI is easy for me.	0.9090
	EOU3*	My interaction with BelRAI is clear and understandable.	0.8965
	EOU5*	It is easy for me to become skilful at using BelRAI.	0.9074
	EOU6*	I find BelRAI easy to use.	0.8782
**ATUT**	A3	I like the idea of using BelRAI.	0.9174
	IM3	I have fun using BelRAI.	0.9600
	AF2*	Working with BelRAI is fun.	0.9351
	Affect1*	I like working with BelRAI.	0.9447
**SI**	SF2*	The senior management of this organisation has been helpful in the use of BelRAI.	0.7663
	SF3	My supervisor is very supportive of the use of BelRAI for my job.	0.9298
	SF4*	In general, the organisation has supported the use of BelRAI.	0.9439
**FC**	PBC1	I have control over using BelRAI.	0.9276
	PBC2*	I have the resources necessary to use BelRAI.	0.8756
	PBC3*	I have the knowledge necessary to use BelRAI.	0.9049
	PBC4	Given the resources, opportunities and knowledge it takes to use BelRAI, it is easy for me to use BelRAI.	0.8203
**SE**	SE4*	Using BelRAI, I could complete the job or task if I had seen someone else using it before trying it myself.	0.7972
	SE5	Using BelRAI, I could complete the job or task if I could call someone for help if I got stuck.	0.8373
	SE6*	Using BelRAI, I could complete the job or task if someone else had helped me get started.	0.8448
	SE9	Using BelRAI, I could complete the job or task if someone showed me how to use it first.	0.8586
**ANX**	ANX1*	I feel apprehensive about using BelRAI.	0.9315
	ANX2*	It scares me to think that I could lose a lot of information using BelRAI by hitting the wrong key.	0.8081
	ANX3*	I hesitate to use BelRAI for fear of making mistakes I cannot correct.	0.8046
	ANX4*	BelRAI is somewhat intimidating to me.	0.8054
**BI**	BI1*	I intend to use BelRAI in the next 3 months.	0.9776
	BI2*	I predict I would use BelRAI in the next 3 months.	0.9708
	BI3*	I plan to use BelRAI in the next 3 months.	0.9614

PE: Performance expectancy; EE: Effort expectancy; ATUT: Attitude towards using technology; SI: Social influence; FC: Facilitating conditions; SE: Self-efficacy; ANX: Anxiety; BI: Behavioural intention.

All questionnaire items were measured using a 7-point Likert agreement scale ranging from “Strongly disagree” to “Strongly agree”. All constructs were modelled using reflective indicators. The items with an asterisk were selected for inclusion in the final UTAUT model in the study of Venkatesh et al. [46]. We refer to the same study for an explanation with regard to the abbreviations and the non-relevant items.

Table 3 summarizes the results for the items comprising our model. As an evaluation of the internal consistency reliability (ICR) of each construct, the values for Cronbach’s alpha (CA) exceed 0.70 the minimal cut-off as recommended by Nunnally [83] and Nunnally et al. [84]. PLS analysis also provides average variance extracted (AVE) and composite reliability (CR). For each construct, AVE exceeds 0.50 and CR exceeds 0.70, which are the recommended thresholds by Fornell et al. [85]. These values denote adequate reliability of the survey instrument.

**Table 3:** The measurement model: reliability results

**Construct**	**Mean**	**Std dev**	**Cronbach’s alpha**	**Construct CR**	**Construct AVE**
**PE**	3.4	1.4	0.98	0.982	0.915
**EE**	4.1	1.3	0.92	0.943	0.806
**ATUT**	3.6	1.4	0.96	0.968	0.883
**SI**	4.4	1.3	0.86	0.914	0.781
**FC**	4.6	1.4	0.91	0.934	0.780
**SE**	4.8	1.0	0.86	0.902	0.697
**ANX**	2.5	1.2	0.88	0.905	0.704
**BI**	5.0	1.7	0.97	0.979	0.941

In accordance with the study of Venkatesh et al. [46] we operationalised the constructs in our UTAUT model by using the highest-loading items from each of the respective scales.

Std Dev: Standard deviation; CR: Composite reliability; AVE: Average variance extracted.

To establish construct validity, PLS examines convergent and discriminant validity of the scale. In this study, each measurement item of the model loads with a t-value above 1.96 on its latent construct. This indicates that each measurement item correlates strongly with its theoretical pre-specified construct. The p-values of the t-values are all significant at the 0.001 alpha protection level, so convergent validity is confirmed^d^. Discriminant validity is established because: 1) the correlation of the latent variable scores with the measurement items shows an appropriate pattern of loadings; the measurement items have high loadings (> 0.70) on their theoretically assigned construct and low loadings (< 0.30) on other constructs. However, the items of the attitude towards using technology construct cross-loaded on PE; 2) the square root of AVE^e^ of each construct (represented by the diagonal values in bold) is significantly higher than the correlations between constructs (represented by the off-diagonal values) in the adjoining columns and rows of Table 4.

**Table 4:** The measurement model: inter-construct correlation matrix with square root of AVE of each construct

	**PE**	**EE**	**ATUT**	**SI**	**FC**	**SE**	**ANX**	**BI**
**PE**	**0.957**							
**EE**	0.499	**0.898**						
**ATUT**	0.795	0.536	**0.940**					
**SI**	0.280	0.394	0.314	**0.778**				
**FC**	0.160	0.606	0.240	0.495	**0.883**			
**SE**	0.033	0.272	0.169	0.215	0.276	**0.835**		
**ANX**	−0.189	−0.387	−0.256	−0.201	−0.390	−0.203	**0.839**	
**BI**	0.165	0.361	0.261	0.368	0.466	0.361	−0.277	**0.970**

Off-diagonal values represent correlations between constructs.

Diagonal values (bold) are the square root of AVE of each construct.

Discriminant validity is confirmed if diagonal values are significantly higher than off-diagonal values.

### The structural model

To test the structural model, PLS estimates path coefficients for each hypothesised path using bootstrapping (100 samples). Path coefficients for each hypothesised path are shown in Table 5 together with the corresponding t-values^f^.

**Table 5:** The structural model: path coefficients, t-values and p-values

	**PE**	**EE**	ATUT*	**SI**	**FC**	SE*	ANX*	**BI**
**Path coefficient**	−0.0390	−0.0010	0.1260	0.1370	**0.2870**	**0.2180**	−0.0690	0.0000
**t-value**	0.3947	0.0131	1.4945	1.8268	**3.6666**	**3.1904**	1.3671	0.000
**p-value**	0.69	0.99	0.14	0.07	**0.00**	**0.00**	0.17	1.00

Constructs with an asterisk were dropped from the final UTAUT model in the study of Venkatesh et al. [46].

Facilitating conditions proved to have the most significant influence on behavioural intention to use BelRAI, followed by self-efficacy. The other constructs have no significant influence on behavioural intention.

Hypothesis 1 is not supported since performance expectancy or the degree to which an individual believes that using the system will help him or her to achieve increases in job performance has no significant influence on behavioural intention (t-value = 0.3947, p-value = 0.69).

In the same manner, hypothesis 2 is not supported since neither effort expectancy nor the degree of ease associated with the use of the system has a significant relationship with behavioural intention (t-value = 0.0131, p-value = 0.99).

In parallel with this there is no significant connection between social influence (the degree to which an individual perceives that important others believe he or she should use the new system) and behavioural intention (t-value > 1.8268, p-value = 0.07) hence not consistent with hypothesis 3.

In accordance with hypothesis 4, facilitating conditions (the degree to which an individual believes that an organisational and a technical infrastructure exists to support use) have a positive effect on behavioural intention to use BelRAI (t-value = 3.6666, p-value = 0.00). This is in contrast with the findings of Venkatesh et al. [46]. They state that facilitating conditions have no significant influence on behavioural intention in the presence of effort expectancy.

Hypothesis 5 is not supported since anxiety described as evoking anxious or emotional reactions when it comes to using a system has no significant influence on behavioural intention (t-value = 1.3671, p-value = 0.17). This affirms the removal of anxiety from the final UTAUT model [46].

Self-efficacy or the judgement of one’s ability to use a system has a positive effect on behavioural intention (t-value = 3.1904, p-value = 0.00), hence hypothesis 6 is supported. This is all the more noteworthy since self-efficacy was dropped from the final UTAUT model in the original study [46].

Finally, hypothesis 7 is not supported since attitude towards using new technology defined as an individual’s overall affective reaction to using a system has no significant influence on behavioural intention (t-value = 1.4945, p-value = 0.14). This affirms the removal of attitude from the final UTAUT model [46].

**Figure 2:**
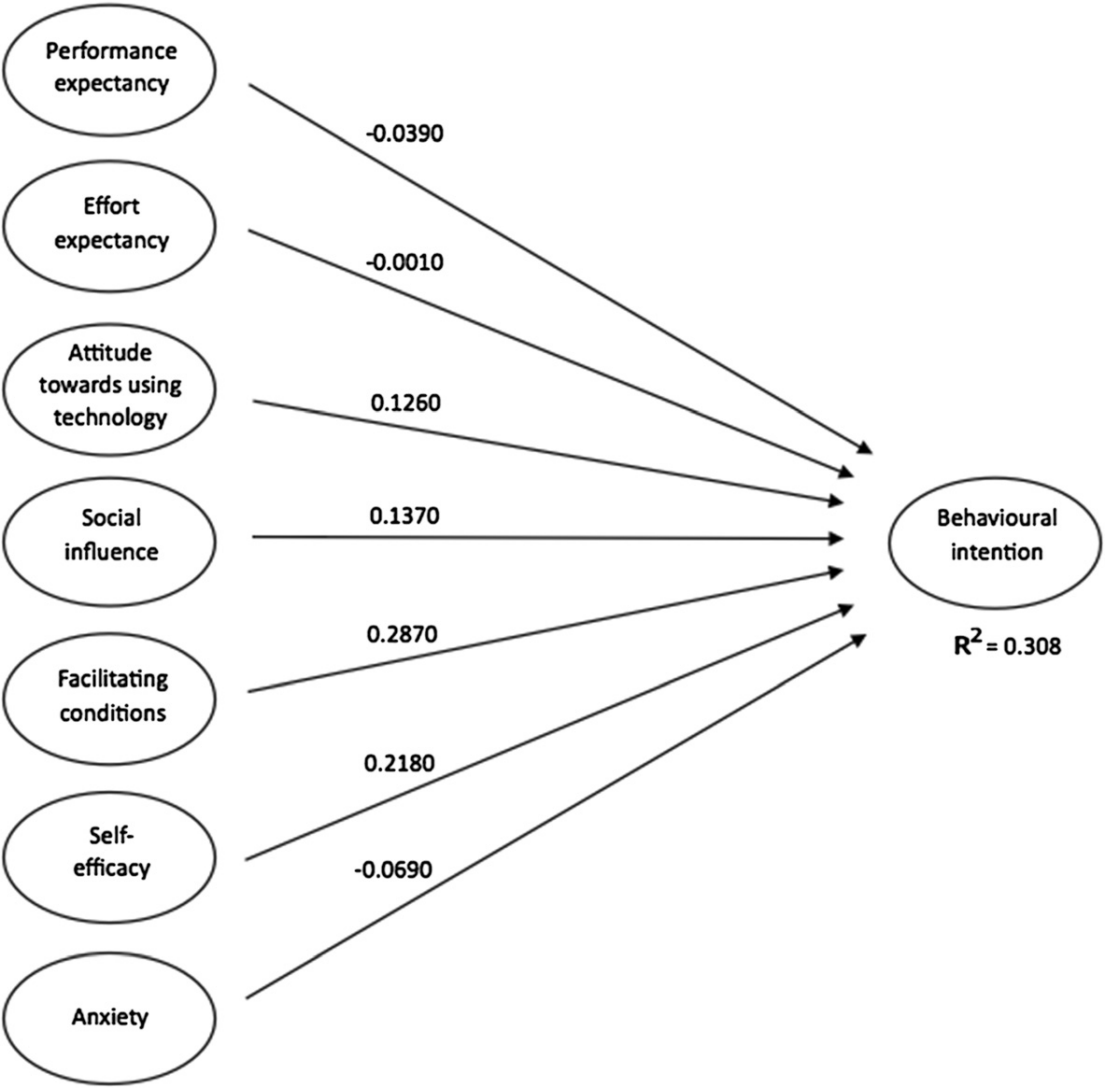
Model testing results.

To ascertain how well the model fits the hypothesised relationship, PLS generates the square of the correlation coefficient (R^2^) for the dependent variable behavioural intention in the model. The R^2^ value for behavioural intention (0.308) indicates that the ‘direct effects only’ model explains 30.8% of the variance in behavioural intention to use BelRAI (Figure 2).

## Discussion

This paper describes a study concerning the utilisation of the UTAUT model to provide an explanation on professional caregivers’ acceptance of the BelRAI web application. Given the fact that UTAUT was originally developed outside the healthcare context, we built upon this framework to complement prior related research on technology acceptance and use [45,48,58,59] by exploring the applicability of the UTAUT model in the specific BelRAI healthcare project - home care, residential care and acute hospital care for older and disabled people.

This study shows that performance expectancy, effort expectancy and social influence do not have a significant influence on behavioural intention as shown in the original UTAUT study [46] and other related research on technology acceptance and use [45,48,58,59]. However, behavioural intention is influenced by facilitating conditions associated with the presence of the resources and the knowledge required to use the system, the degree of compatibility of the new system with other systems in use and the availability of assistance in the case of system difficulties. Similarly, self-efficacy associated with the possibility to complete a task using the new information system with only the built-in help facility, having the possibility to call someone for help and having enough time to practice, has a significant influence on behavioural intention to use the BelRAI web application.

These results seem to be in line with the findings by Declercq et al. [68]. Inadequate computer equipment (hardware, software, internet connection) causes frustration among users, is counterproductive, and constitutes significant barriers to accepting the new technology. At the same time it is important to achieve the right balance between confidentiality and accessibility. The BelRAI web application meets the requirements set by the Belgian Commission for the Protection of Privacy, and the privacy and security standards have been positively evaluated by the users. However, rigid security regulations also result in access procedures that are complex and time-consuming, as a result of which they negatively influence the application’s user-friendliness. Occasionally, when it is possible to get around the security measures (by exchanging the eIDs, for instance), a result will be reached that is opposite to the one intended.

In any case, there is need for high quality education, both internally and externally. The expectations for external education and support can strongly depend on the context or setting. In one setting there might be a need for practical education, whereas in another a rather theoretical training might be appropriate. In an initial phase the training can be organised per setting, so that each setting gets specific information based on its needs. During a subsequent phase, the training groups can be composed rather heterogeneously in order to put an emphasis on the interaction and collaboration between different settings. This is particularly appreciated by home care caregivers since they hardly get in touch with other caregivers active in both home care and other care settings. Also, the periods in between different training phases should be long enough in order to enable caregivers to acquire experience and give feedback. The group sizes and the number of teachers must be in tune with one another so that teachers can adopt a problem-solving approach. The internal training sessions, organised by the organisations themselves, should be very practical. For an organisation, it is appropriate to appoint a reference person who can hold the position of internal trainer and act as a contact person. Assigning some staff members to work on the project can facilitate a full-time follow-up and implementation. The learning period does not end after the final training session. In order to be able to fluently use new technological tools, each caregiver should pass through a post-training learning process. Moreover, each caregiver should be given the opportunity to gain sufficient experience in a variety of situations. In addition to the technological aspect, the content (assessment) as such constitutes an indispensable part of the learning process. Frequent and intensive exercises are required in order to have good command of all applications. However, frequent staff changes can seriously disturb the learning process.

Time is an important resource. In practice, a high workload, a lack of personnel, high client turnover, and the presence of acute problems (acute hospitals) turn out to be significant barriers standing in the way of implementing new information technology. In order to improve efficiency and avoid an increased workload, the degree of integration of existing information technology (e.g., electronic patient records) with new technologies is decisive in the success of implementation. It is in everyone’s interest that everything can happen in a user-friendly and safe way, without technical failures and with a minimum of intermediate steps. Integrated technology leads to fewer difficulties and will be accepted and used more rapidly.

During the implementation of new technologies, it is also important for active caregivers not to feel alone, but rather to feel supported in times of doubt by the care team, the (middle) management and other caregivers involved. Other factors that can contribute to a successful implementation are a help desk, a wiki website, and the possibility of being able to work in a quiet environment. Moreover, the collaboration process can be influenced by the physical proximity of various disciplines: people working within the same organisation collaborate more easily than people employed at different sites.

Although the use of the BelRAI application was mandatory, it is plausible that the behavioural intention of professionals is influenced by the short-term nature of this project. Simulating mandatory use of new information technology in a public healthcare setting is not straightforward. Therefore, since the future professional value of BelRAI is not guaranteed, performance expectancy may not have influenced behavioural intention. Similarly, effort expectancy may not have a significant relationship with behavioural intention because people tend not to exert great effort during a pilot project. Social influence may not have an impact on behavioural intention because of a difference in attitude between caregivers and typical technology users. Future study designs should disentangle these effects inside the UTAUT framework to understand user acceptance in the context of a permanent implementation. The measurement of behavioural intention to use new information technology could be expanded to take into account the emotional disposition of professional caregivers. Ultimately, when new information technology is implemented on a large scale, professionals should be looking forward to using it.

## Conclusions

The practical implications of this research are that good-quality software, interoperability and compatibility with other information systems, easy access to computers, a degree of computer literacy, training facilities, built-in and online help and ongoing IT support are critical influencing factors in stimulating the behavioural intention to use new technology. These findings can be used by policy makers to maximise the acceptance and the success of new technology initiatives.

For researchers, this study indicates that a bottom-up approach should be preferred when building upon the UTAUT model. The conclusions of the original UTAUT study [46] with regards to the item and scale construction should not be copied blindly across different information systems. In the specific context of BelRAI, self-efficacy is an important influencing factor of behavioural intention while this factor is not retained in the original study.

Our elaboration of the UTAUT model is limited since no information is available about the actual use of the BelRAI web application. Other research should encompass variables that relate to actual use (e.g. frequency and duration) in order to investigate the influence of behavioural intention. In this research it is not possible to gain insight into non-response bias and to know who is cooperative and who is not. It is likely that respondents who did not complete the survey have a more negative attitude towards new technology. Future research should provide insight into the characteristics of the entire study population. Future research should also take into account experience with the system in a longitudinal evaluation and examine the effects of age, gender, healthcare context, profession, computer literacy and specific training sessions not only related to behavioural intention but also to actual usage of the system. To better understand healthcare professionals’ acceptance of BelRAI, more research is needed to generalise the findings of this feasibility project to a future, broad implementation.

## Endnotes

^a^Since 2006, different government-funded feasibility studies concerning the implementation of interRAI assessment instruments at a national level were carried out by an interuniversity team of the KU Leuven (Elderly Care Research Unit at LUCAS& Center for Health Services and Nursing Research) and the Université de Liège (Département des Sciences de la Santé Publique) [68,87-91].

^b^85 questionnaire items related to the UTAUT constructs were measured: Performance expectancy (PE), 24 items; Effort expectancy (EE), 10 items; Attitude towards using technology (ATUT), 14 items; Social influence (SI), 9 items; Facilitating conditions (FC), 11 items; Self-efficacy (SE), 10 items; Anxiety (ANX), 4 items; Behavioural intention (BI), 3 items.

^c^Selection based on item loadings is consistent with recommendations in the psychometric literature [84]. “This approach favours building a homogenous instrument with high internal consistency but could sacrifice content validity by narrowing domain coverage” – items from some of the models not being represented in some of the core constructs [46].

^d^The p-value of this t-value should be significant at the 0.05 alpha protection level [80].

^e^By the latest version of PLS-Graph 03.00 build 1126 of 2003 AVEs are generated automatically using bootstrapping as a resampling procedure. The AVE measures the variance captured by a latent construct being the shared (or explained) variance. The square root of AVE of each construct should be much larger than its variance shared with any of the other constructs in the model and should be at least 0.50. Conceptually, it is equivalent to saying that the correlation between the construct and its measurement items should be larger than its correlation with the other constructs [45,76,80].

^f^The corresponding t-values suggest significance of the coefficients (t-values > 1.96, significance level p < 0.05) [80]. PLS generates the square of the correlation coefficient (R^2^) for each dependent construct in the model. R^2^ is interpreted as the percentage of shared (or explained) variance and thus represents the proportion of variance in the dependent constructs which can be explained by the independent ones [45,77].

## Abbreviations

UTAUT: Unified theory of acceptance and use of technology; ICT: Information and communication technologies; IT: Information technology; IS: Information systems; SCT: Social cognitive theory; TRA: Theory of reasoned action; TPB: Theory of planned behaviour; MPCU: Model of PC utilization; TAM: Technology acceptance model; C-TAM-TPB: Combined TAM and TPB; IDT: Innovation diffusion theory; MM: Motivational model; PE: Performance expectancy; EE: Effort expectancy; SI: Social influence; FC: Facilitating conditions; BI: Behavioural intention; ANX: Anxiety; SE: Self-efficacy; ATUT: Attitude towards using technology; SEM: Structural equation modelling; LISREL: Linear structural relations; PLS: Partial least squares; SAS: Statistical analysis system; ICR: Internal consistency reliability; CA: Cronbach’s alpha; AVE: Average variance extracted; CR: Composite reliability; Std Dev: Standard deviation.

## Competing interests

The authors declare that they have no competing interests.

## Authors’ contributions

VD, VB and DA are involved in the study design and critically reviewed and approved the final manuscript. VD drafted the manuscript. All authors read and approved the final manuscript.

## Pre-publication history

The pre-publication history for this paper can be accessed here:

http://www.biomedcentral.com/1472-6947/13/129/prepub
